# Expression of collagenases-1 and -3 and their inhibitors TIMP-1 and -3 correlates with the level of invasion in malignant melanomas

**DOI:** 10.1038/sj.bjc.6690417

**Published:** 1999-05

**Authors:** K Airola, T Karonen, M Vaalamo, K Lehti, J Lohi, A-L Kariniemi, J Keski-Oja, U K Saarialho-Kere

**Affiliations:** Department of Dermatology, Helsinki University Central Hospital, Meilahdentie 2, Helsinki, FIN-00250, Finland; Department of Virology, The Haartman Institute, Haartmaninkatu 3, Helsinki, FIN-00014, Finland

**Keywords:** MMP, cell invasion, angiogenesis, gelatinase

## Abstract

Since proteolysis of the dermal collagenous matrix and basement membranes is required for local invasive growth and early metastasis formation of cutaneous melanomas, we have analysed the activities/expression levels of certain metalloproteinases in melanomas and cultured melanoma cells by in situ hybridization and Northern analysis. In addition to collagenases-1 and -3 that have been implicated in invasive growth behaviour of various malignant tumours, we analysed the levels of 72-kDa gelatinase and its activators MT1-MMP and TIMP-2 in cultured melanoma cells. The lesions examined included three cases of lentigo maligna and 28 cases of Clark grade I–V melanomas. The premalignant as well as the grade I tumours were consistently negative for collagenase-1 and -3 and TIMP-1 and -3. The collagenases were predominantly expressed in the cancer cells of Clark grade III and IV tumours. TIMP-1 and -3 were abundantly expressed in the cancer and/or stromal cells of grade III and IV melanomas, while TIMP-2 protein was detected also in melanomas representing lower invasive potential. Northern analysis of seven melanoma cell lines showed that the expression of collagenase-1 and TIMPs-1 and -3 was associated with 72-kDa gelatinase positivity. All melanoma cell lines were positive for MTI-MMP and TIMP-2 mRNAs. Our results suggest that overexpression of collagenases-1 and -3 and TIMPs -1 and -3 is induced during melanoma progression. Expression of TIMPs may reflect host response to tumour invasion in an effort to control MMP activity and preserve extracellular matrix integrity. © 1999 Cancer Research Campaign


*British Journal of Cancer* (1999) **80**(5/6), 733–743
© 1999 Cancer Research Campaign
Article no. bjoc.1998.0417

### Expression of collagenases-1 and -3 and their inhibitors TIMP-1 and -3 correlates with the level of invasion in malignant melanomas

**K Airola[1], T Karonen[1,2], M Vaalamo[1], K Lehti[2], J Lohi[2], A-L Kariniemi[1], J Keski-Oja[1,2] and UK Saarialho-Kere[1]**

[1]Department of Dermatology, Helsinki University Central Hospital, Meilahdentie 2, FIN-00250 Helsinki, Finland; [2]Department of Virology, The Haartman Institute, Haartmaninkatu 3, PO Box 21, FIN-00014, Helsinki, Finland

**Summary** Since proteolysis of the dermal collagenous matrix and basement membranes is required for local invasive growth and early metastasis formation of cutaneous melanomas, we have analysed the activities/expression levels of certain metalloproteinases in melanomas and cultured melanoma cells by in situ hybridization and Northern analysis. In addition to collagenases-1 and -3 that have been implicated in invasive growth behaviour of various malignant tumours, we analysed the levels of 72-kDa gelatinase and its activators MT1-MMP and TIMP-2 in cultured melanoma cells. The lesions examined included three cases of lentigo maligna and 28 cases of Clark grade I–V melanomas. The premalignant as well as the grade I tumours were consistently negative for collagenase-1 and -3 and TIMP-1 and -3. The collagenases were predominantly expressed in the cancer cells of Clark grade III and IV tumours. TIMP-1 and -3 were abundantly expressed in the cancer and/or stromal cells of grade III and IV melanomas, while TIMP-2 protein was detected also in melanomas representing lower invasive potential. Northern analysis of seven melanoma cell lines showed that the expression of collagenase-1 and TIMPs-1 and -3 was associated with 72-kDa gelatinase positivity. All melanoma cell lines were positive for MTI-MMP and TIMP-2 mRNAs. Our results suggest that overexpression of collagenases-1 and -3 and TIMPs -1 and -3 is induced during melanoma progression. Expression of TIMPs may reflect host response to tumour invasion in an effort to control MMP activity and preserve extracellular matrix integrity.

**Keywords:** MMP; cell invasion; angiogenesis; gelatinase

Tumour invasion and metastasis is a multistep process that requires loss of growth control, tumour cell locomotion and attachment, proteolysis of the extracellular matrix (ECM) and tumour cell colony formation in the target organ for metastasis (Stetler-Stevenson et al, 1993). Proteolysis of the ECM is principally mediated by two classes of enzymes: members of the plasminogen activator/plasmin system and the matrix metalloproteinase (MMP) family. The MMPs include 16 structurally related, zinc-dependent endopeptidases that are divided into subfamilies of collagenases, stromelysins, gelatinases, membrane-type MMPs and novel MMPs according to their structure and substrate specificity (Birkedal-Hansen et al, 1993; Llano et al, 1997; Pendas et al, 1997). The active enzymes are specifically inhibited by TIMPs (tissue inhibitor of metalloproteinases). As a group, MMPs are capable of degrading essentially all components of the ECM, and increased MMP activity has been correlated with the progression of malignant tumours of various origins, including the lung, prostate, breast, colon and skin (reviewed by Stetler-Stevenson et al, 1993).

Malignant melanomas are highly invasive and metastatic tumours that are becoming increasingly prevalent worldwide. The most important prognostic factors in patients without metastases are the level of invasion and tumour thickness. The depth of invasion can be classified to various histological levels according to Clark et al (1969), whereas Breslow (1970) measures tumour thickness from the top of the epidermal granular layer to the deepest extension of the tumour. Other factors contributing to unfavourable prognosis include lymph node metastases, axial location, male sex, high age, wide diameter of the lesion and ulceration (Lever and Schaumburg-Lever, 1990).

Overexpression of 72-kDa (MMP-2) and 92-kDa (MMP-9) gelatinases has been correlated with the invasive phenotype in both melanoma cell lines and melanoma cells in vivo (Höyhtyä et al, 1990; Montgomery et al, 1993; McDougall et al, 1995; Brooks et al, 1996; Väisänen et al, 1996). Expression of collagenase-1 (MMP-1) and stromelysin-1 (MMP-3) has also been reported in metastatic melanoma cell lines (Brown et al, 1990; Montgomery et al, 1993). Recently, invasion of melanoma cells through type I and IV collagens and matrigel was shown to be suppressed by antisense RNA for MMP-1 (Durko et al, 1997). The ability of TIMP-1 and -3 to inhibit tumour growth has been observed by overexpressing these MMP-inhibitors in melanoma cell lines (Khokha, 1994; Anand-Apte et al, 1996). According to a recent report, TIMP-2 overexpression reduces the invasion and angiogenesis-inducing properties of some melanoma cells in vivo and protects them from apoptosis (Valente et al, 1998). Furthermore, TIMP-2 is important in melanoma biology in the MMP-2 activation cascade. MT1-MMP (MMP-14) binds TIMP-2 specifically, generating an MTI-MMP–TIMP-2 complex, which, in turn, functions as a receptor for the MMP-2 proenzyme (Strongin et al, 1995; Butler et al, 1998).

We have previously found that expression of the novel MMP, collagenase-3 (MMP-13), is up-regulated during malignant transformation of non-melanoma skin tumours (Airola et al, 1997; Johansson et al, 1997). Both MMPs-1 and -13 efficiently degrade

*Received 10 September 1998*
*Revised 3 December 1998*
*Accepted 3 December 1998*

*Correspondence to:* UK Saarialho-Kere

**Table 1** Characteristics of patients and expression of MMPs and TIMPs in cutaneous melanomas

| Clark | Breslow (mm) | Subtype | Sex/age | Loc/Metast. | MMP-1 | MMP-13 | TIMPs | | | MMP-2 | MT1-MMP |
|---|---|---|---|---|---|---|---|---|---|---|---|
| | | | | | | | -1 | -3 | -2 | | |
| I | n.d. | ss | F/63 | Palm/no | – | – | – | – | +ca,s | | – |
| I | n.d. | ss | F/50 | leg/n.d. | – | – | – | – | – | – | – |
| I | n.d. | ss | F/27 | leg/no | – | – | – | – | +ca | +ca,s | – |
| I | 0.4 | ss | M/58 | side/no | – | – | – | – | | | +s |
| II | 0.2 | ss | M/52 | chest/no | – | – | – | – | | – | +s |
| II | 0.3 | ss | M/51 | shoulder/no | – | – | – | – | +ca,s | ++s | ++s |
| II | 0.4 | ss | F/25 | chest/no | – | – | – | – | ++s | +s | |
| II | 0.45 | ss | M/53 | neck/n.d. | +ca | – | – | – | +s | – | – |
| II | 0.5 | ss | F/28 | back/n.d. | – | – | +s | – | ++ca,s | – | – |
| III | 0.6 | ss | F/78 | face/no | – | – | – | – | | | |
| III | 0.7 | ss | M/45 | chest/no | +ca | – | – | – | +ca,s | | |
| III | 0.7 | ss | F/55 | leg/n.d. | +ca | +ca | +++ca,s | +s | +s | +s | |
| III | 0.9 | n | F/36 | ear/n.d. | ++ca | – | +ca,s | +ca,s | +s | +s | |
| III | 0.95 | ss | M/59 | back/no | – | – | – | – | – | – | |
| III | 1.1 | ss | F/44 | back/no | – | – | – | – | | | |
| III | 1.7 | n | F/57 | arm/yes | – | – | ++ca | +ca | | | |
| III | 2.0 | n | F/79 | face/no | +ca | +ca | +s | – | +s | | |
| III | 2.6 | n | M/39 | abdomen/no | +++ca | +ca | +++ca,s | ++ca,s | | | |
| IV | 2.1 | n | M/58 | back/yes | ++ca | +++ca | +++ca | +++ca | +s | +s | +s |
| IV | 2.7 | n | M/93 | ear*/no | – | – | +++ca,s | ++ca,s | | | |
| IV | 2.4 | ss | M/51 | genitals/no | +ca | – | +ca | (+)ca | +ca | (+)ca | +ca,s |
| IV | 3.0 | n | M/71 | arm/no | +ca | (+)ca | +ca,s | (+)s | ++ca | – | +ca |
| IV | 3.5 | n | M/86 | leg*/no | +s | – | +++s | ++s | +ca | | |
| IV | 3.5 | n | M/62 | neck/no | ++ca | +ca | +s | ++ca,s | ++s | +s | +ca,s |
| IV | 4.0 | n | F/33 | back/no | – | – | ++ca,s | | | | |
| IV | 5.0 | n | M/74 | back*/n.d. | – | – | +s | +ca,s | ++ca,s | ++s | ++s |
| IV | 6.0 | n | F/83 | arm*/n.d. | ++ca | ++ca | +++ca,s | +ca | | | |
| V | 6.5 | n | F/84 | arm/no | +ca | – | +ca | (+)ca | ++ca | | +s |

ss: superficially spreading; n: nodular; n.d.: not determined; ca: expression in tumour cells; s: expression in stromal cells; *ulcerated tumour type I, II and III collagens. Furthermore, MMP-13 is gelatinolytic and type IV collagenolytic (Knäuper et al, 1996, 1997), and MMP-1 has also some activity against full-length type IV collagen (Collier et al, 1988). Our objective was to determine the expression patterns and cellular localization of these MMPs during melanocytic tumour progression. Furthermore, as imbalance between TIMPs and MMPs is an important factor in tumour invasion, the expression levels of collagenase inhibitors TIMP-1 and -3 were also examined. Our results suggest that the induction of MMPs-1 and -13 and their tissue inhibitors is a late event in melanocytic tumour progression, and that these enzymes are involved in the regulation of melanoma invasion.

### MATERIALS AND METHODS

### Tissue specimens

Formalin-fixed, paraffin-embedded specimens were obtained from the Department of Dermatopathology, Helsinki University Central Hospital, Helsinki, Finland. Invasion levels of the melanomas were determined by Clark's classification: level I with confinement of the malignant melanoma cells to the epidermis and its appendages; level II with extension to the papillary dermis so that only a few melanoma cells extended to the interface between the papillae and reticular dermis; level III with filling the papillary dermis until the boundary between the papillary and the reticular dermis; level IV with invasion of the reticular dermis; and level V with invasion of the subcutaneous fat (Clark et al, 1969). Furthermore, tumour thickness was measured according to Breslow (tumours less than 0.76 mm thick represent low metastatic risk while those more than 1.5 mm are of high risk; Breslow, 1970). The samples included three lentigo malignas, four grade I, five grade II, nine grade III, nine grade IV and one grade V malignant melanomas, as assessed by an experienced dermatopathologist (A-LK).

### RNA probes

The production and specificity of the 550-bp MMP-1, 490-bp MMP-13, 313-bp TIMP-1 and 635-bp TIMP-3 antisense probes have been described earlier in detail (Saarialho-Kere et al, 1992; Johansson et al, 1997; Airola et al, 1998). The cDNAs were transcribed in vitro using a commercial kit (Promega Corp., Madison, WI, USA) and labelled with $^{35}$S-UTP, as previously described (Saarialho-Kere et al, 1993).

### cDNA probes

Full-length human cDNA for MMP-2 was given by Dr G Goldberg, Department of Dermatology, Washington University (Collier et al, 1988). A 699 bp cDNA probe corresponding to bases 884–1582 of MT1-MMP sequence was generated by reverse transcription polymerase chain reaction (RT-PCR) from HT-1080 cDNA (Lohi and Keski-Oja, 1995). A 537 bp cDNA fragment was used for detecting TIMP-2 (provided by Dr G Goldberg). Rat glyceraldehyde 3-phosphate dehydrogenase (GAPDH) cDNA (Forth et al, 1985) was used to standardize the loading of RNA.

 *© Cancer Research Campaign 1999*

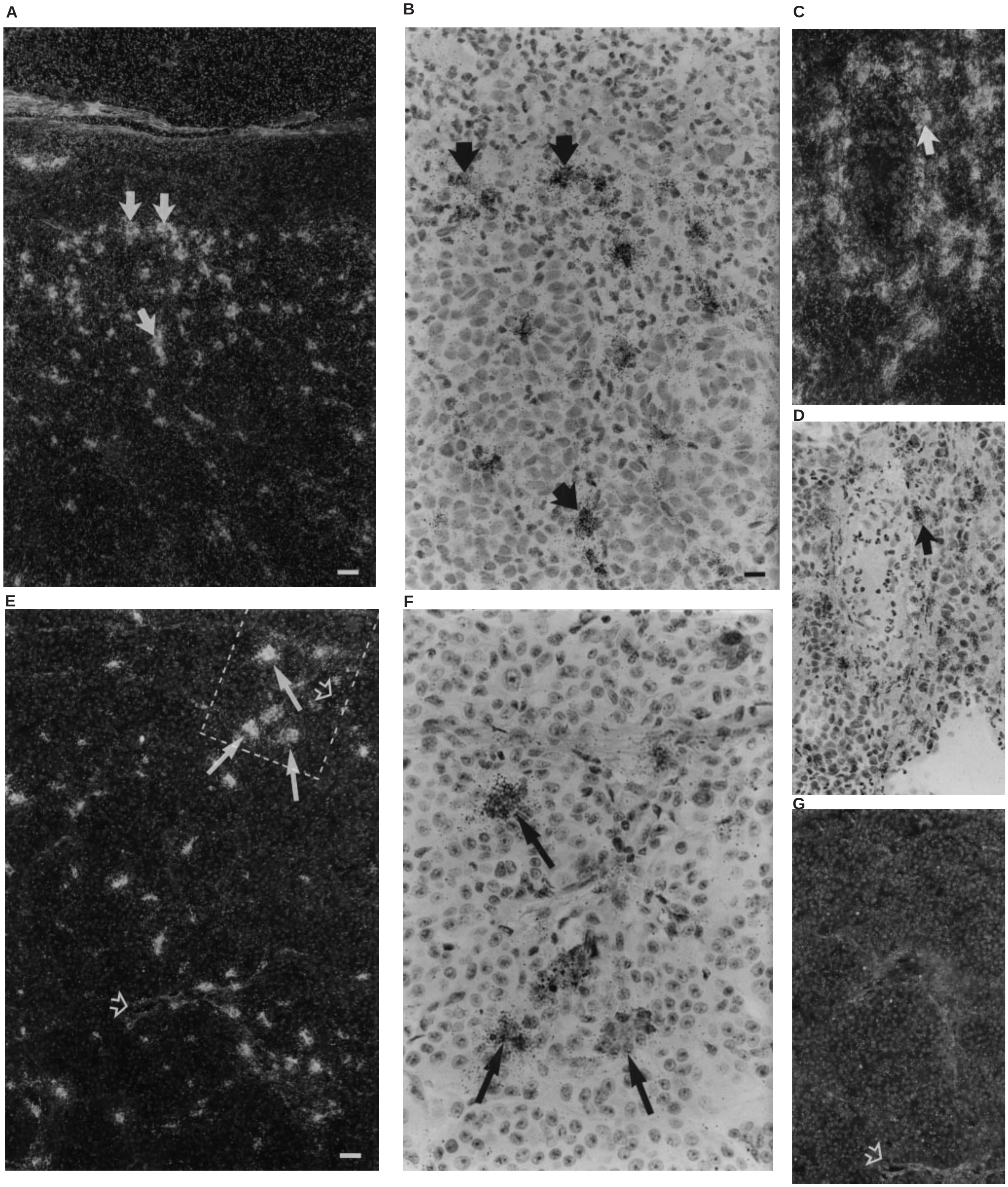

**Figure 1** Expression of MMPs-1 and -13 by melanoma cells adjacent to the stroma. (**A**) Dark-field image of MMP-1 mRNA in tumour cells (arrows) of grade III melanoma (magnification 100 ×) and (**B**) high magnification (400 ×) of the MMP-1 positive cells. (**C**) Another area of the same tumour (200 ×). Note the collagenous stroma adjacent to the MMP-1 expressing cells (arrows). (**D**) Corresponding bright-field image. (**E**) MMP-13 mRNA in tumour cells (arrows) of grade IV melanoma. (**F**) Higher magnification of the MMP-13-positive cells. (**G**) Same specimen hybridized with MMP-1 probe, indicating differential localization of expression of the two collagenases. Open arrows depict corresponding locations (**E**, **G**). Scale bar: **A**, 40 μm; **C**, **D**, **E**, 20 μm; **B**, **F**, 10 μm

*© Cancer Research Campaign 1999*

### In situ hybridization

Following deparaffinization and rehydration, sections were pretreated with 1 mg ml$^{-1}$ of proteinase K and washed in 0.1 M triethanolamine containing 0.25% acetic anhydride. Subsequently, sections were hybridized with probes ($2.5–5 \times 10^4$ cpm ml$^{-1}$ of hybridization buffer) and washed under stringent conditions, including treatment with RNAase, as described (Saarialho-Kere et al, 1993). Autoradiography was carried out for 10–30 days. All samples were processed in at least two experiments, and were independently examined by two experienced investigators. The signals were scored arbitrarily as follows: (–) no detectable signal, (+) specific signal in a few cells, (++) specific signal in a moderate number of cells, and (+++) specific signal in a high number of cells.

### Immunohistochemistry

Immunostaining was performed using the avidin–biotin–peroxidase complex technique (Saarialho-Kere et al, 1993). Aminoethylcarbazole was used as chromogenic substrate. Monoclonal antibodies included anti-MMP-2 (clone 42-5D11, Oncogene Research Products, Cambridge, MA, USA), anti-MT1-MMP (clone 114-6G6, Oncogene Research Products) and anti-TIMP-2 (clone 67-4H11, Fuji Chemicals, Japan). The tissues were counterstained with haematoxylin and controls were performed with pre-immune mouse ascites fluid.

### Cell cultures and Northern hybridization analysis

The majority of the melanoma cell lines have been isolated from melanoma metastases and most of them were established at the Wistar Institute (WM, Wistar melanoma). Human fibrosarcoma HT-1080 cells (CCL-121, American Type Culture Collection, Rockville, MD, USA) and melanoma cell lines Bowes, G361, WM793, WM852, WM164, WM165 and WM239 were cultivated in Eagle's minimal essential medium (MEM) (Gibco BRL, Gaithersburg, MD, USA), containing 10% heat inactivated fetal calf serum, 100 IU ml$^{-1}$ penicillin, and 50 mg ml$^{-1}$ streptomycin. Confluent cultures were incubated under serum-free conditions for 6 h and subsequently with 40 nM PMA (phorbol myristate acetate) for 24 h as indicated. RNA was purified by the guanidium thiocyanate method (Chomczynski and Sacchi, 1987), and 15 μg of total RNA was electrophoresed on a 0.8% formaldehyde-agarose gel and transferred to a Zeta probe filter (Bio-Rad, Richmond, CA, USA) and immobilized by UV cross-linking. The filters were hybridized to probes labelled with $^{32}$P-dCTP (Amersham) by the random priming method (Pharmacia LKB, Uppsala, Sweden). Hybridization (42°C, 24 h) and subsequent washing were performed under stringent conditions. Stripping, when needed, was performed in 0.1 × saline–sodium citrate (SSC), 0.5% sodium dodecyl sulphate (SDS) at 100°C for 10 min.

### Assays for gelatinolytic activity

Conditioned media from HT-1080 and melanoma cells were assayed by gelatin zymography essentially as described (Lohi et al, 1992). Samples of conditioned medium were dissolved in non-reducing Laemmli sample buffer and separated by electrophoresis using discontinuous 3.5:7% polyacrylamide gels containing 1 mg ml$^{-1}$ gelatin (Laemmli, 1970). After electrophoresis the gels were washed twice with 50 mM Tris–HCl buffer pH 7.6, containing 5 mM calcium chloride, 1 μM zinc chloride, 2.5% Triton X-100 (v/v) for 15 min to remove SDS, followed by brief rinsing in washing buffer without Triton X-100. The gels were incubated at 37°C for 1–2 days in 50 mM Tris–HCl buffer containing 5 mM calcium chloride, 1 μM zinc chloride, 1% Triton X-100, 0.02% $NaN_3$, pH 7.6. The digestion was terminated by 10% acetic acid followed by staining with Coomassie brilliant blue R250 and destaining with 10% acetic acid, 10% methanol. Zones of enzymatic activity were seen as negatively stained bands.

### Reversed gelatin zymography

Samples of HT-1080 and melanoma cells were treated as described above (assays for gelatinolytic activity). Aliquots of concentrated conditioned medium (corresponding to the amount secreted by $10^4$ cells) were analysed by electrophoresis using discontinuous 4:12% polyacrylamide gels containing 0.7 mg ml$^{-1}$ gelatin and 35 ng ml$^{-1}$ 72-kDa gelatinase (Chemicon International, Inc., Temecula, CA, USA). After electrophoresis the gels were washed, incubated for 40–50 h, stained and destained as described above (assays for gelatinolytic activity).

### RESULTS

### MMPs-1 and -13 are expressed by invasive melanoma cells

To assess whether MMPs -1 and -13 and TIMPs -1 and -3 are expressed in melanomas in vivo, 28 samples were studied using in situ hybridization. Lentigo malignas and grade I tumours were consistently negative for both MMP-1 and -13 mRNA, while 1/5 grade II, 5/9 grade III, and 6/9 grade IV tumours expressed MMP-1 (Table 1). MMP-13 mRNA was detected in 3/9 grade III and 4/9 grade IV tumours (Table 1). Both collagenases were expressed by tumour cells (Figure 1). MMP-1 mRNA was more abundant and detected specifically at the edges of tumour nodules that were surrounded by areas of connective tissue (Figure 1 C,D), while MMP-13 mRNA was detected more centrally within the tumour (Figure 1 E,F). Generally, tumour front facing the dermis was negative for both collagenases.

### TIMPs-1 and -3 are abundantly expressed in grade III and IV melanomas

Since the activity of collagenases is effectively controlled and locally restricted by TIMPs, we analysed next the expression of TIMPs-1 and -3 in the specimens. TIMP-1 and -3 mRNAs were frequently detected in the grade III and IV melanomas in both tumour and stromal cells (Table 1). Five of nine of grade III, 9/9 of grade IV tumours and 1/1 grade V tumour expressed TIMP-1 mRNA, and 4/9 of grade III, 8/9 of grade IV and 1/1 grade V tumours TIMP-3 mRNA. TIMP-1 expression was more abundant at the tumour-stromal front (Figure 2 A–D). In addition to tumour cells and fibroblasts, TIMP-3 mRNA was detected in endothelial cells within connective tissue septae (Figure 2 E,F). Only two of the tumours had metastasized during the follow-up period (1–7 years). No follow-up information was available from seven out of 28 patients (Table 1). The mRNA expression patterns of collagenases or TIMPs did not reveal any clear differences between the metastasized and localized tumours (Table 1).

 © Cancer Research Campaign 1999

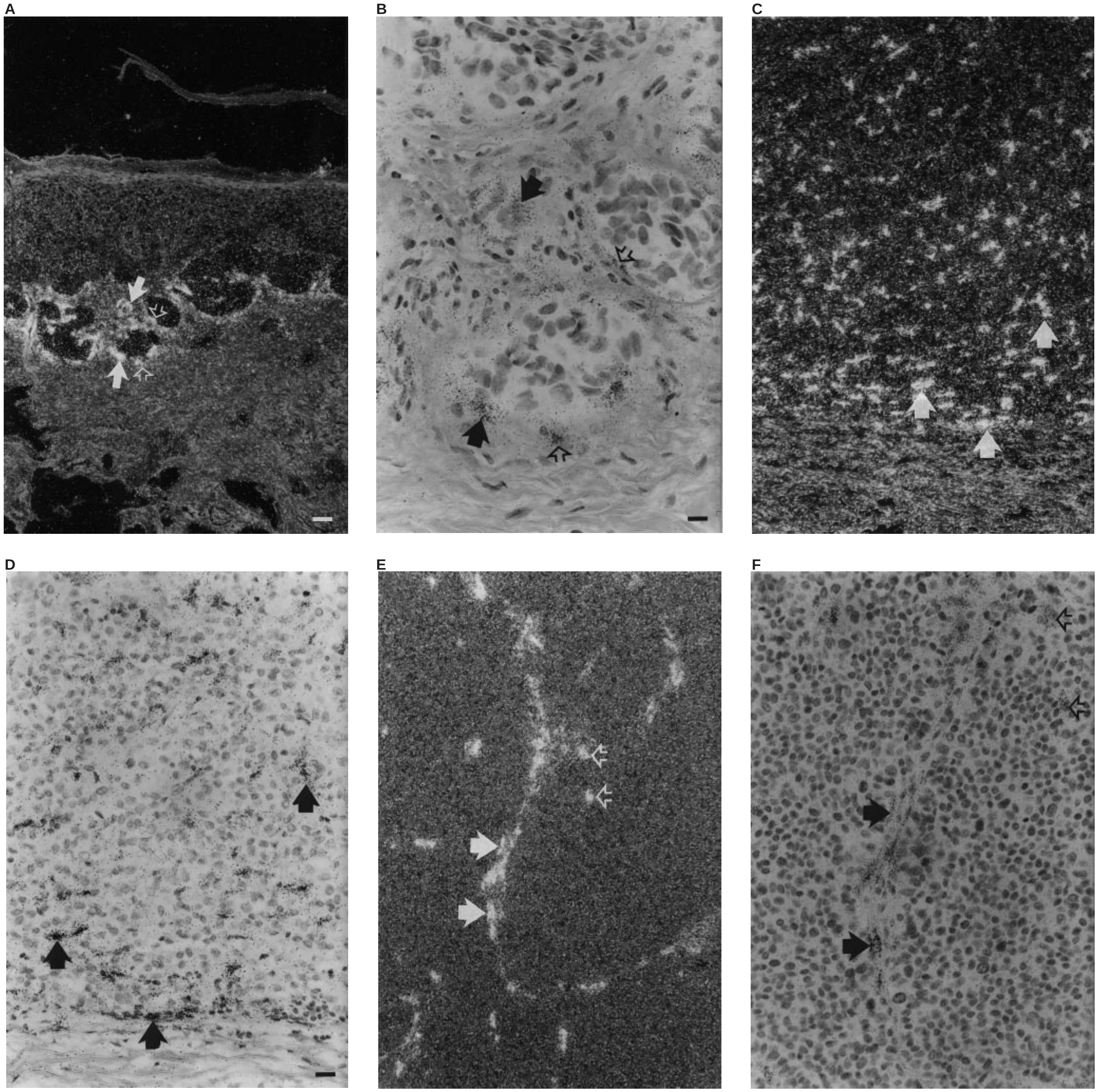

**Figure 2** Expression of TIMP-1 and -3 mRNAs. (**A**) TIMP-1 mRNA in tumour cells (arrows) and stromal cells (open arrows) in grade III melanoma. (**B**) Higher magnification bright-field of the same specimen. (**C**) Grade IV melanoma with abundant signal for TIMP-1 mRNA in tumour cells (arrows) at the tumour-stromal front. (**D**) Higher magnification bright-field of the tumour front facing dermis. (**E**) Dark-field image of TIMP-3 mRNA expression in grade IV tumour (arrow = stromal cell, open arrow = tumour cell). (**F**) High-power bright-field image of TIMP-3-expressing cells. Arrows indicate endothelial cells within connective tissue septae. Scale bar: **A**, **C**, **E**, 40 μm; **D**, **F**, 20 μm; **B**, 10 μm

### Northern hybridization analysis

To verify the ability of melanoma cells to express collagenases and TIMPs in cell culture, seven different human melanoma cell lines (see Figure 3 for details) were grown to confluency, changed to serum-free culture medium and treated with PMA (40 mM) for 24 h followed by RNA extraction and Northern hybridization analysis. MMP-1 mRNA was expressed in 3/7 (Figure 3) unstimulated cell lines, all of which also expressed MMP-2 abundantly. No MMP-13 mRNA was detected, whereas control human lung fibroblasts (CCL-137) and HaCaT keratinocytes expressed detectable levels of this enzyme (data not shown). All TIMPs were frequently expressed: TIMP-1 and -2 in 7/7 and TIMP-3 in 5/7 cell lines. Expression of TIMP-1 was, however, more abundant than that of TIMP-3. Treatment of the cells by PMA generally stimulated MMP and TIMP expression (Figure 3).

© Cancer Research Campaign 1999

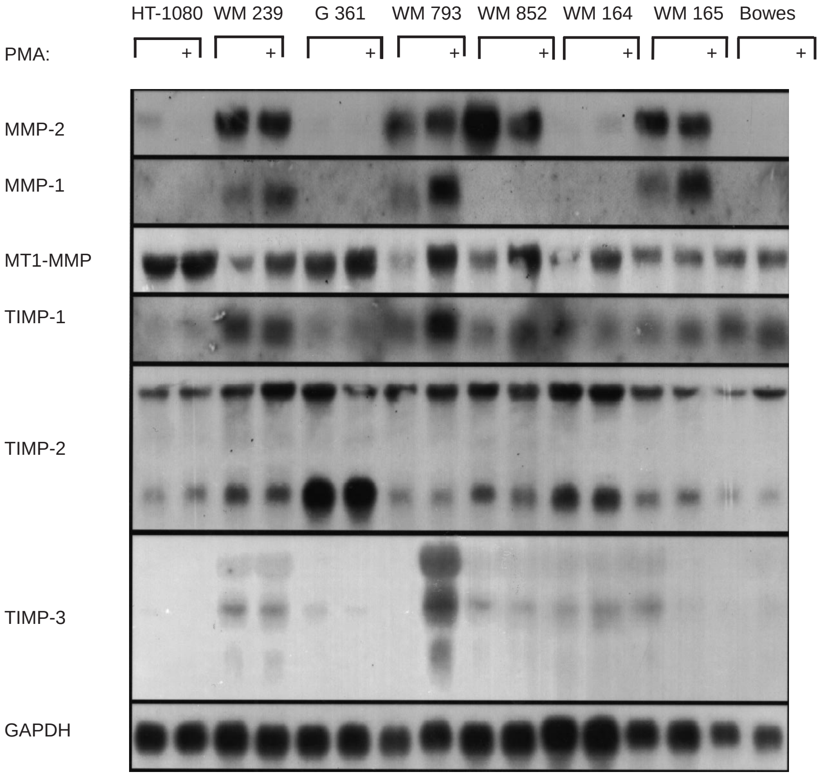

**Figure 3** Northern hybridization analysis for MMP-2, MMP-1, MT1-MMP, TIMP-1, -2 and -3 mRNAs in HT-1080 and the different melanoma cell lines (designations given on the Figure) treated with PMA as indicated for 24 h. Because of the variation of the levels of MMP-2 mRNA some cell lines appear negative in this exposure. Longer exposures gave faint bands indicating that MMP-2 is expressed in all melanoma lines studied (compare with zymographic analysis). GAPDH mRNA levels served as internal standard

### MT1-MMP and MMP-2 are expressed by cultured melanoma cells

Overexpression of MT1-MMP has been associated with invasiveness of cancer cells, evidently via activation of MMP-2 (Sato et al, 1994). The levels of MT1-MMP mRNA in melanoma cell lines were therefore assayed by Northern hybridization. HT-1080 human fibrosarcoma cells were used as a control (Lohi et al, 1996). MT1-MMP was expressed at a considerable level in all melanoma cell lines and in four cell lines (WM239, WM793, WM852 and WM164) its expression was increased about twofold by PMA (Figure 3). mRNA levels of MMP-2 were exceptionally high in some melanoma cell lines (WM239, WM793, WM852 and WM165), while in three other melanoma cell lines (G361, WM164, Bowes) MMP-2 was expressed at a level comparable to that of HT-1080 cells (Figure 3). Interestingly, the cell lines G361 and WM164, that were negative for both MMP-1 and -2 mRNAs, have been established from primary non-metastatic melanoma cell lines (Peebles et al, 1980; Iliopoulos et al, 1989). Cell lines expressing both MMP-1 and -2 (WM239, WM165, ML852) have been established from melanoma metastases (Herlyn et al, 1985*a*, 1985*b*). However, since Bowes cell line established from a metastatic melanoma (Opdenakker et al, 1983; Rifkin DB, personal communication) was MMP-1 negative and WM793 grown from human primary melanoma (Herlyn et al, 1985*b*) positive for both MMPs-1 and -2, no fully consistent conclusions between MMP-1 and -2 positivity and invasiveness can be drawn.

### TIMP-2 protein is expressed in melanomas independent on their Clark classification

Based on our results on melanoma cell lines, we also conducted an immunohistochemical study for MMP-2, MT1-MMP and TIMP-2 in a subset of melanomas, including samples from all Clark grades (Table 1). TIMP-2 protein was expressed in stromal (fibroblasts and endothelial cells in cancer stroma and surrounding the tumour) or cancer cells in 17/19 samples (Figure 4 A–C) and, unlike TIMPs-1 and -3, its expression was abundant also in melanomas with low invasive potential (Table 1). MMP-2 protein was detected mainly in stromal cells bordering the tumour in 9/15 samples (Figure 4D). MT1-MMP was most abundantly expressed in cancer cells of Clark grade IV tumours (Table 1; Figure 4 E,F).

### Melanoma cells secrete MMP-2 in latent form

To correlate the MT1-MMP expression to MMP-2 activation, culture medium of melanoma cells and HT-1080 cells from a similar experiment were analysed by gelatin zymography. Untreated HT-1080 cells produced, in addition to MMP-9, the MMP-2 mainly in the latent form. It was efficiently processed to the active 62-kDa form in cultures treated with PMA (Figure 5A). On the contrary, all melanoma cell lines secreted MMP-2 in latent form only, and no lower molecular weight forms corresponding to the activated 62-kDa form were seen, even in the culture medium of cells treated with PMA (Figure 5A).

 © Cancer Research Campaign 1999

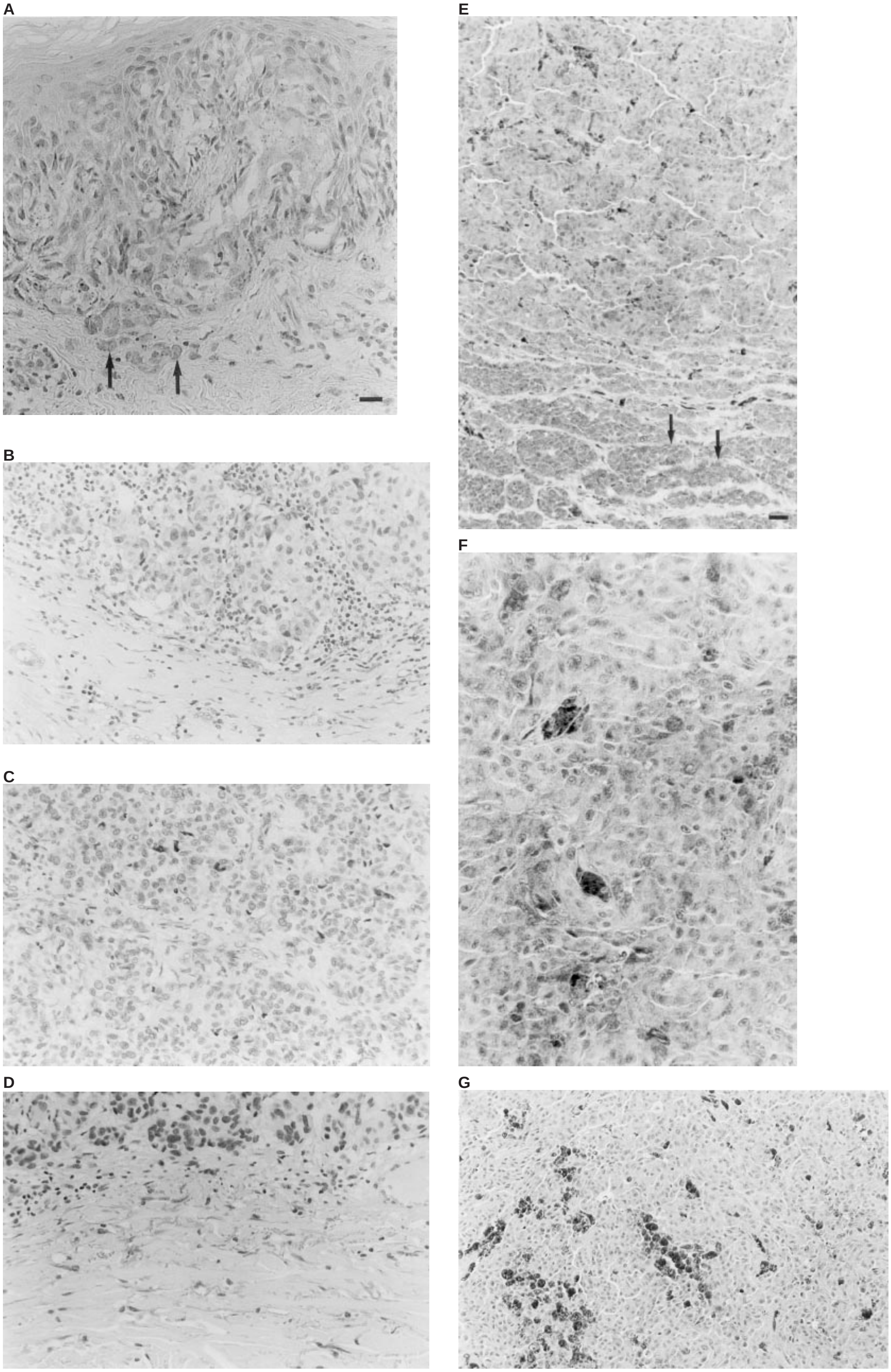

**Figure 4** Immunostaining for TIMP-2 is positive in invasive melanoma cells (arrows) in Clark grade I (**A**) and IV (**B**) melanomas. (**C**) TIMP-2 staining is detected in fibroblasts and endothelial cells inside the tumour in the same specimen. (**D**) MMP-2 protein is detected in fibroblasts in the stroma surrounding the tumour. (**E**) Clark grade IV melanoma with positive immunostaining for MT1-MMP in melanoma cells at the invasive front of the tumour (arrows). (**F**) A higher magnification of MT1-MMP positive cells inside the tumour. (**G**) Negative control. AEC was used as chromogenic substrate and haematoxylin as counterstain. Scale bar: **A**, **B**, **C**, **D**, **F**, 20 μm; **E**, **G**, 40 μm

*© Cancer Research Campaign 1999*

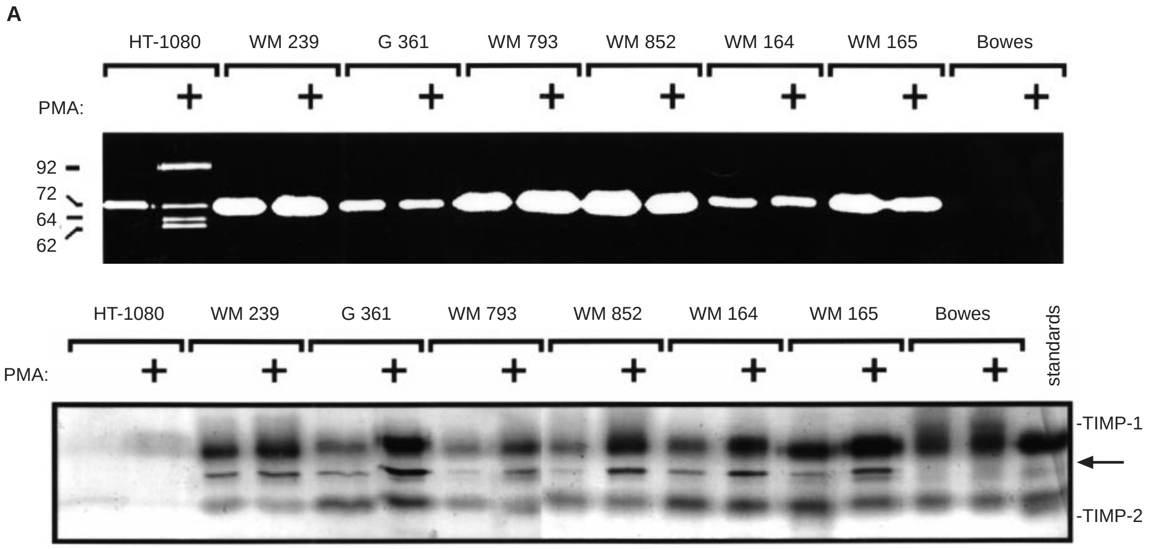

**Figure 5** (**A**) Cultured melanoma cells secrete only the latent form of MMP-2. Confluent cultures of HT-1080 and melanoma cells were treated with PMA under serum-free conditions for 24 h as shown in the Figure (see Methods). Aliquots of conditioned medium (corresponding to the amount secreted by $10^4$ cells for melanomas and $5 \times 10^4$ cells for HT-1080) were analysed by gelatin zymography using 7% SDS-PAGE. The level secreted by Bowes cells was barely detectable (in accordance with Northern analysis) but positive. (**B**) All melanoma cell lines produce TIMP-1 and -2, the levels of TIMP-1 being more inducible by PMA than the levels of TIMP-2. Aliquots of concentrated conditioned medium from cells incubated with or without PMA were analysed by reversed gelatin zymography. An arrow indicates the migration of a third TIMP activity band, probably corresponding to TIMP-3

### Reversed gelatin zymography

To analyse the levels of TIMP-1 and -2 in the melanoma cell lines, we carried out reverse zymography assays (see Methods). All melanoma cell lines produced TIMP-1 and -2 (Figure 5B). The levels of TIMP-1 (31 kDa) were slightly more clearly inducible by PMA than the levels of TIMP-2 (21 kDa). The levels of the TIMPs were relatively low in control HT-1080 cells. All melanoma lines except Bowes secreted also gelatinase inhibitory activity of 25–27 kDa, most probably corresponding to TIMP-3. This was inducible by PMA treatment (Figure 5B).

### DISCUSSION

Proteolytic activity produced either by tumour cells or surrounding stromal cells is fundamental to invasion and metastasis of malignant tumours, including melanomas. Expression of gelatinases and members of the plasminogen activator/plasmin system, in particular, has been proposed to contribute to the aggressive and metastatic growth of malignant melanomas (Mueller, 1996). In this work we observe that melanoma cells express MMPs-1, -2, -14 and TIMPs-1, -2 and -3 both in vivo and vitro. To our knowledge, this is the first report on the tissue localization of MMPs-1 and -13 as well as TIMPs-1, -2 and -3 in cutaneous malignant melanomas. We describe that the induction of MMPs-1 and -13 and their tissue inhibitors is a late event in melanocytic tumour progression, and correlates with the invasion of cutaneous melanomas in vivo. These results substantiate the role of collagenases in melanoma invasion, and demonstrate that high levels of TIMP expression paradoxically associate with an advanced tumour stage and subsequent poor prognosis.

MMP-1 is frequently expressed by stromal cells of non-melanoma skin cancers (Kähäri and Saarialho-Kere, 1997). MMP-13 was originally cloned from human breast carcinomas (Freije et al, 1994), and the mRNA is expressed predominantly in tumour cells of squamous cell carcinomas of the head and neck and basal cell carcinomas (Airola et al, 1997; Johansson et al, 1997). In contrast to frequent overexpression of MMP-1 in the stroma of various cancers, both collagenases were produced by malignant melanoma cells themselves. In melanomas, the production of MMPs-2 and -9 has been reported during the initial radial growth phase and during early invasive phase, as the tumour cells penetrate the basement membrane and migrate into the papillary dermis (Väisänen et al, 1996; van den Oord et al, 1997). In contrast, members of the plasminogen activator/plasmin system are predominantly expressed during the highly invasive vertical growth phase (de Vries et al, 1994), similarly to MMPs-1 and -13. It is likely that temporally and spatially differentially regulated expression of proteinases allows selective degradation of ECM proteins and efficient tumour cell migration. Thus, expression of multiple invasion-associated proteinases may represent an important feature of a tumour that has acquired the ability for metastatic growth.

Expression of neither collagenases nor TIMPs could be associated with clinical metastases, since only two tumours had metastasized during the follow-up period. However, expression of MMP-1 by tumour cells adjacent to connective tissue septae containing blood vessels may be associated with early metastatic events, e.g. tumour cell migration towards the vessel wall. The role of MMP-13, produced by individual, centrally located tumour cells, may be equally associated with proteolytic degradation of the connective tissue. Alternatively, MMP-13 may be involved in other processes such as apoptosis, as demonstrated previously in basal cell carcinomas (Airola et al, 1997). In contrast to the in vivo results, none of the melanoma cell lines tested expressed MMP-13. As demonstrated by Uria et al (1997) by using co-cultures of fibroblasts and breast cancer cell lines, interactions between both cell types are essential in modulating MMP-13 expression by human fibroblasts. This suggests that expression of MMP-13 may require the collaboration of several cell types and thus lack of stromal cells or the different pericellular environment may explain the absence of

 *© Cancer Research Campaign 1999*

MMP-13 in melanoma cell lines. The number of cells containing MMP-13 mRNA in the lesions studied was clearly smaller than those having signal for collagenase-1 or TIMPs. Nevertheless, the in vivo data presented here promotes the association of MMP-13 expression with malignant transformation.

The expression of TIMPs-1 and -3 was associated with progressive invasion in malignant melanomas. Since angiogenesis is required for expansion of solid tumours, the enhanced expression may be related to the anti-angiogenic activity reported for both inhibitors (Johnson et al, 1994; Anand-Apte et al, 1997). Expression of TIMP-3 in endothelial cells within the tumour supports this hypothesis. Interestingly, in vivo overexpression of TIMP-3 in certain melanoma cell lines is associated with apoptosis (Ahonen et al, 1998) and we cannot exclude that some TIMP-3 mRNA positive tumour cells were actually undergoing programmed cell death. TIMP-1 was predominantly expressed at the invasion front, suggesting rather a role in inhibition of proteolysis at the advancing tumour edge. Since MMP-1 and -13 mRNAs were not frequently detected at this area, the inhibitory activity of TIMP-1 may be directed towards other proteinases, such as stromelysins or MMP-9.

It has been proposed that the cell surface concentrations of TIMPs are crucial for the ability of MT1-MMP to activate MMP-2. In HT-1080 fibrosarcoma cells we have observed efficient processing of MT1-MMP and activation of MMP-2 in response to PMA (Lohi et al, 1996; Lehti et al, 1998). The levels of TIMPs were low in the conditioned medium of HT-1080 cells, while they were clearly seen in the melanomas. TIMP-1 was especially prominent, but it is not known to participate in or interfere with MMP-2 activation. TIMP-2 levels were slightly inducible by PMA, as well as TIMP-3.

We have attempted to correlate here also the expression of certain MMPs and their inhibitors to those of established cell lines. We observed expression of MT1-MMP, MMP-2 and TIMPs-1, -2 and -3, which was in most cases induced by the phorbol ester PMA. Expression of MMP-13 was, however, not noted. The correlation remained thus inconclusive. This is probably due to several reasons. Cultivation is known to affect the expression of several genes and to select subpopulations of cells that may not reflect the status of the original tumour. In addition, the connective tissue as well as inflammation characteristics of melanomas, are missing from the surroundings of cultured cells, which modifies the expression of MMPs.

MT1-MMP is able to cleave type I–III collagens, gelatin, fibronectin and laminin and to activate MMP-13 (Murphy and Knäuper, 1997). Its enhanced expression in various tumours suggests that MT1-MMP is an important enzyme in cell invasion. Lack of MMP-2 activation even in those melanoma cell lines, which express high levels of MT1-MMP, suggests that in culture these cells may need some exogenous factors like TIMP-2 or contact with e.g. collagen for MMP-2 activation (Strongin et al, 1995; Butler et al, 1998). We found that TIMP-2 was expressed at a fairly similar level in all melanoma cell lines and HT-1080 cells (Figure 3). Consequently, lack of MMP-2 activation by melanoma cell lines cannot be attributed to differential regulation of TIMP-2 expression. On the other hand, cultured melanoma cells may not possess the capability present in in vivo melanomas because of different pericellular environment and lack of stromal cells. Our previous results (Lohi et al, 1995) indicate that although overexpression of MT1-MMP is in some cell lines enough to induce MMP-2 activating activity, the expression of MT1-MMP per se is not sufficient to cause this.

No previous histological data are available on the expression of MT1-MMP and TIMP-2 in melanocytic tumours. Väisänen et al (1996) detected MMP-2 immunoreactivity in 50% of their melanomas. We found MMP-2 mostly in stromal cells, which may be explained by a limited number of cases analysed as well as different antibodies. TIMP-2 was detected in melanoma cells, fibroblasts or in endothelial cells in the majority of tumours examined, also associated with a low invasion level. MT1-MMP expression was more often detected in cancer cells in tumours with high invasive potential. In a recent study on cancer cell types other than melanoma (Deryugina et al, 1997), cell transfectants characterized by partial MMP-2 activation and relatively low levels of MMP-2 in its active form were most invasive. This implies that a balance of MT1-MMP expression, amounts of cell surface-bound and soluble TIMP-2, levels of TIMP-2-free MMP-2 proenzyme and activated MMP-2, as well as occupancy of matrix adhesion receptors, is critical to tumour cells becoming highly invasive.

An imbalance between cell surface associated proteinases and their inhibitors (higher concentrations of MMPs and lower of TIMPs) has been traditionally implicated in tumour expansion. However, increased expression of TIMP-1 and -3 in highly malignant tumours of the gastrointestinal tract and breast has also been demonstrated (Yoshiji et al, 1996; Airola et al, 1997; Mimori et al, 1997; Powe et al, 1997), in addition to malignant melanomas in this report. These data raise the question whether TIMPs possess dual functions in the regulation of tumour growth and invasion. In addition to suppressing pericellular proteolysis and neovascularization, they may promote tumour cell proliferation through growth factor-like activity (Fong et al, 1996; Gomez et al, 1997) at some stage of tumour progression. Thus, there may be different mechanisms involved between cancer growth and invasion of early and advanced melanomas, which requires particular attention when considering therapeutic intervention with synthetic TIMP analogues.

### ACKNOWLEDGEMENTS

We thank Dr Veli-Matti Kähäri for the MMP-13 and TIMP-3 cDNAs and Ms Alli Tallqvist for her excellent technical assistance. This work was supported by the Paulo Foundation, The Academy of Finland, Finnish Cancer Foundation (KL, JL, JK-O), and the Helsinki University Central Hospital.

### REFERENCES

Ahonen M, Baker AH and Kähäri V-M (1998) Adenovirus-mediated gene delivery of tissue inhibitor of metalloproteinases-3 inhibits invasion and induces apoptosis in melanoma cells. *Cancer Res* **58**: 2310–2315

Airola K, Johansson N, Kariniemi A-L, Kähäri V-M and Saarialho-Kere UK (1997) Human collagenase-3 is expressed in malignant squamous epithelium of the skin. *J Invest Dermatol* **109**: 225–231

Airola K, Ahonen M, Johansson N, Heikkilä P, Kere J, Kähäri V-M and Saarialho-Kere UK (1998) Human TIMP-3 in expressed during fetal development, hair growth cycle and cancer progression. *J Histochem Cytochem* **46**: 437–448

Anand-Apte B, Bao L, Smith R, Iwata K, Olsen BR, Zetter B and Apte SS (1996) A review of tissue inhibitor metalloproteinases-3 (TIMP-3) and experimental analysis of its effect on primary tumor growth. *Biochem Cell Biol* **74**: 853–862

Anand-Apte B, Pepper MS, Voest E, Montesano R, Olsen B, Murphy G, Apte SS and Zetter B (1997) Inhibition of angiogenesis by tissue inhibitor metalloproteinase-3. *Invest Ophthal Visual Sci* **38**: 817–823

*© Cancer Research Campaign 1999* 

Birkedal-Hansen H, Moore WGI, Bodden MK, Windsor LJ, Birkedal-Hansen B, DeCarlo A and Engler J A (1993) Matrix metalloproteinases: a review. *Crit Rev Oral Biol Med* **4**: 197–250

Breslow A (1970) Thickness, cross-sectional areas and depth of invasion in the prognosis of cutaneous melanoma. *Ann Surg* **172**: 902–908

Brooks PC, Strömblad S, Sanders LC, von Schalscha, TL, Aimes RT, Stetler-Stevenson WG, Quigley JP and Cheresh DA (1996) Localization of matrix metalloproteinase MMP-2 to the surface of invasive cells by interaction with integrin αvβ3. *Cell* **85**: 683–693

Brown PD, Levy AT, Margulies IMK, Liotta LA and Stetler-Stevenson WG (1990) Independent expression and cellular processing of $M_r$ 72 000 type IV collagenase and interstitial collagenase in human tumorigenic cell lines. *Cancer Res* **50**: 6184–6191

Butler G, Butler MJ, Atkinson SJ, Will H, Tamura T, Schade van Westrum S, Crabbe T, Clements J, d'Ortho M-P and Murphy G (1998) The TIMP-2 membrane type 1 metalloproteinase 'receptor' regulates the concentration and efficient activation of progelatinase A. *J Biol Chem* **273**: 871–880

Chomczynski P and Sacchi N (1987) Single-step method of RNA isolation by acid guanidium thiocyanate-phenol-chloroform extraction. *Anal Biochem* **162**: 156–159

Clark WH Jr, From L and Bernardino EH (1969) Histogenesis and biologic behavior of primary human malignant melanoma of the skin. *Cancer Res* **29**: 705–727

Collier IE, Wilhelm SM, Eisen AZ, Marmer BL, Grant GA, Selzer JL, Kronberger A, He C, Bauer EA and Goldberg GI (1988) H-*ras* oncogene-transformed human bronchial epithelial cells (TBE-1) secrete a single metalloproteinase capable of degrading basement membrane collagen. *J Biol Chem* **263**: 6579–6587

Deryugina E, Luo G-X, Reisfeld RA, Bourdon MA and Strongin A (1997) Tumor cell invasion through Matrigel is regulated by activated matrix metalloproteinase-2. *Anticancer Res* **17**: 3201–3210

Durko M, Navab R, Shibata HR and Brodt P (1997) Suppression of basement membrane type IV collagen degradation and cell invasion in human melanoma cells expressing an antisense RNA for MMP-1. *Biochim Biophys Acta* **1356**: 271–280

Fong KW, Kida Y, Zimmerman PV and Smith PJ (1996) TIMP-1 and adverse prognosis in non-small cell lung cancer. *Clin Cancer Res* **2**: 1369–1372

Forth P, Marty L, Piechaczyk M, El Sabrouty S, Dani C, Jeanteur P and Blanchard JM (1985) Various rat adult tissues express only one major mRNA species from the glyceraldehyde-3-phosphate-dehydrogenase multigenic family. *Nucleic Acids Res* **13**: 1431–1422

Freije JMP, Díez-Itza I, Balbín M, Sánchez M, Blasco R, Tolivia J and López-Otín C (1994) Molecular cloning and expression of collagenase-3, a novel human matrix metalloproteinase produced by breast carcinomas. *J Biol Chem* **269**: 16766–16733

Gomez DE, Alonso DF, Yoshiji H and Thorgeirsson UP (1997) Tissue inhibitors of MMPs: structure, regulation and biological functions. *Eur J Cell Biol* **74**: 111–122

Herlyn B, Balaban G, Bennicelli J, DuPont IV G, Halaban R, Herlyn D, Elder DE, Maul GG, Steplewski Z, Nowell PC, Clark WH and Koprowski H (1985*a*) Primary melanoma cells of the vertical growth phase: similarities to metastatic cells. *J Natl Cancer Inst* **74**: 283–239

Herlyn M, Thurin J, Balaban G, Bennicelli J, Herlyn D, Elder DE, Bondi E, Guerry D, Nowell P, Clark WH and Koprowski H (1985*b*) Characteristics of cultured human melanocytes isolated from different stages of tumor progression. *Cancer Res* **45**: 5670–5676

Höyhtyä M, Hujanen E, Turpeenniemi-Hujanen T, Thorgeirsson U, Liotta LA and Tryggvason K (1990) Modulation of type-IV collagenase activity and invasive behavior of metastatic human melanoma (A2058) cells in vitro by monoclonal antibodies to type-IV collagenase. *Int J Cancer* **46**: 282–286

Iliopoulos D, Ernst C, Steplewski Z, Jambrosic JA, Rodeck U, Herlyn M, Clark WH, Koprowski H and Herlyn D (1989) Inhibition of metastases of a human melanoma xenograft by monoclonal antibody to the GD2/GD3 gangliosides. *J Natl Cancer Inst* **81**: 440–444

Johansson N, Airola K, Greman R, Kariniemi A-L, Saarialho-Kere U and Kähäri V-M (1997) Expression of collagenase-3 (matrix metalloproteinase-13) in squamous cell carcinomas of the head and neck. *Am J Pathol* **151**: 499–508

Johnson MD, Choi Kim H-R, Chesler L, Tsao-Wu G, Bouck N and Polverini PJ (1994) Inhibition of angiogenesis by tissue inhibitor of metalloproteinase. *J Cell Physiol* **160**: 194–202

Kähäri V-M and Saarialho-Kere U (1997) Matrix metalloproteinases in skin. *Exp Dermatol* **6**: 199–213

Khokha R (1994) Suppression of the tumorigenic and metastatic abilities of murine B16-F10 melanoma cells in vivo by the overexpression of the tissue inhibitor of metalloproteinases-1. *J Natl Cancer Inst* **86**: 299–304

Knäuper V, López-Otín C, Smith B, Knight G and Murphy G (1996) Biochemical characterization of human collagenase-3. *J Biol Chem* **271**: 1544–1550

Knäuper V, Cowell S, Smith B, Lopez-Otin C, O'Shea M, Morris H, Zardi L and Murphy G (1997) The role of C-terminal domain of human collagenase-3 (MMP-13) in the activation of procollagenase-3, substrate specificity, and tissue inhibitor of metalloproteinase interaction. *J Biol Chem* **272**: 7608–7616

Laemmli UK (1970) Cleavage of structural proteins during the assembly of the head of bacteriophage T4. *Nature (Lond)* **227**: 680–685

Lehti K, Lohi J, Valtanen H and Keski-Oja J (1998) Proteolytic processing of membrane-type-1 matrix metalloproteinase is associated with gelatinase A activation at the cell surface. *Biochem J* **334**: 345–353

Lever WF and Schaumburg-Lever G (1990) Benign melanocytic tumors and malignant melanoma. In *Histopathology of the Skin*, Lever WF and Schaumburg-Lever G (eds), pp. 756–805. JB Lippincott: Philadelphia

Llano E, Pendas AM, Knäuper V, Sorsa T, Salo T, Salido E, Murphy G, Simmer JP, Bartlett JD and Lopez-Otin C (1997) Identification and structural and functional characterization of human enamelysin (MMP-20). *Biochemistry* **36**: 15101–15108

Lohi J and Keski-Oja J (1995) Calcium ionophores decrease pericellular gelatinolytic activity via inhibition of 92-kDa gelatinase expression and decrease of 72-kDa gelatinase activation. *J Biol Chem* **270**: 17602–17609

Lohi J, Harvima I and Keski-Oja J (1992) Pericellular substrates of human mast cell tryptase: 72,000 dalton gelatinase and fibronectin. *J Cell Biochem* **50**: 337–349

Lohi J, Lehti K, Westermarck J, Kähäri V-M and Keski-Oja J (1996) Regulation of membrane-type matrix metalloproteinase (MT-MMP) expression by growth factors and the phorbol ester PMA. *Eur J Biochem* **239**: 239–247

McDougall JR, Bani MR, Lin Y, Rak J and Kerbel RS (1995) The 92-kDa gelatinase B is expressed by advanced stage melanoma cells: suppression by somatic cell hybridization with early stage melanoma cells. *Cancer Res* **55**: 4174–4181

Mimori K, Shiraishi T, Fujie T, Baba K, Haraguchi M, Abe R, Ueo H and Akiyoshi T (1997) Clinical significance of tissue inhibitor of metalloproteinase expression in gastric carcinoma. *Br J Cancer* **76**: 531–536

Montgomery AMP, De Clerck YA, Langley KE, Reisfeld RA and Mueller BM (1993) Melanoma-mediated dissolution of extracellular matrix: Contribution of urokinase-dependent and metalloproteinase-dependent proteolytic pathways. *Cancer Res* **53**: 693–700

Mueller BM (1996) Different roles for plasminogen activators and metalloproteinases in melanoma metastasis. *Curr Top Microbiol Immunol* **213**: 65–80

Murphy G and Knäuper V (1997) Relating matrix metalloproteinase structure to function: why the 'hemopexin' domain? *Matrix Biol* **15**: 511–518

van den Oord JJ, Paemen L, Opdenakker G and Wolf-Peeters CD (1997) Expression of gelatinase B and the extracellular matrix metalloproteinase inducer EMMPRIN in benign and malignant pigment cell lesions of the skin. *Am J Pathol* **151**: 665–670

Opdenakker G, Ashino-Fuse H, van Damme J, Billiau A and de Somer P (1983) Effects of 12-O-tetradecanoylphorbol 13-acetate on the production of mRNAs for human tissue-type plasminogen activator. *Eur J Biochem* **131**: 481–487

Peebles PT, Trisch T, Papageorge AG (1980) Isolation of four unusual pediatric solid tumor cell lines. *Ped Res* **12**: 485

Pendas AM, Knäuper V, Puente XS, Llano E, Mattei MG, Apte S, Murphy G and Lopez-Otin C (1997) Identification and characterization of a novel human matrix metalloproteinase with unique structural characteristics, chromosomal location, and tissue distribution. *J Biol Chem* **272**: 4281–4286

Powe DG, Brough JL, Carter GI, Bailey EM, Stetler-Stevenson WG, Turner DR and Hewitt RE (1997) TIMP-3 mRNA expression is regionally increased in moderately and poorly differentiated colorectal adenocarcinoma. *Br J Cancer* **75**: 1678–1683

Saarialho-Kere UK, Chang ES, Welgus HG and Parks WC (1992) Distinct localization of collagenase and tissue inhibitor of metalloproteinases expression in wound healing associated with ulcerative pyogenic granuloma. *J Clin Invest* **90**: 1952–1957

Saarialho-Kere UK, Kovacs SO, Pentland AP, Olerud JE, Welgus HG and Parks WC (1993) Cell-matrix interactions modulate interstitial collagenase expression by human keratinocytes actively involved in wound healing. *J Clin Invest* **92**: 2858–2866

Sato H, Takino T, Okada Y, Cao J, Shinagawa A, Yamamoto E and Seiki M (1994) A matrix metalloproteinase expressed on the surface of invasive tumour cells. *Nature (Lond)* **370**: 61–65

Stetler-Stevenson WG, Liotta LA and Kleiner DE Jr (1993) Extracellular matrix: role of matrix metalloproteinases in tumor invasion and metastasis. *FASEB J* **7**: 1434–1441

Strongin AY, Collier I, Bannikov G, Marmer BL, Grant GA and Goldberg GI (1995) Mechanism of cell surface activation of 72 kDa type IV collagenase. Isolation

 © Cancer Research Campaign 1999

of the activated form of the membrane metalloproteinase. *J Biol Chem* **270**: 5331–5338
Uria JA, Stahle-Bäckdahl M, Seiki M, Fueyo A and Lopez-Otin C (1997) Regulation of collagenase-3 expression in human breast carcinomas is mediated by stromal-epithelial cell interactions. *Cancer Res* **57**: 4882–4888
Valente P, Fassina G, Melchiori A, Masiello L, Cilli M, Vacca A, Onisto M, Santi L, Stetler-Stevenson WG and Albini A (1998) TIMP-2 over-expression reduces invasion and angiogenesis and proptects B 16F10 melanoma cells from apoptosis. *Int J Cancer* **75**: 246–253
de Vries TJ, Quax PHA, Denijn M, Verrijp KN, Verheijen JH, Verspaget HW, Weidle UH, Ruiter DJ and van Muijen GNP (1994) Plasminogen activators, their inhibitors, and urokinase receptor emerge in late stages of melanocytic tumor progression. *Am J Pathol*. **144**: 70–81
Väisänen A, Tuominen H, Kallioinen M and Turpeenniemi-Hujanen T (1996) Matrix metalloproteinase-2 (72 kD type IV collagenase) expression occurs in the early stage of human melanocytic tumour progression and may have prognostic value. *J Pathol* **180**: 283–289
Yoshuji H, Gomez DE and Thorgeirsson UP (1996) Enhanced RNA expression of tissue inhibitor of metalloproteinases-1 (TIMP-1) in human breast cancer. *Int J Cancer* **69**: 131–134

© Cancer Research Campaign 1999
